# MicroRNA-631 deriving from bone marrow mesenchymal stem cell exosomes facilitates the malignant behavior of non-small cell lung cancer via modulating the E2F family of transcription factor 2/phosphatidylinositol 3‐kinase/Akt signaling pathway

**DOI:** 10.1080/21655979.2022.2036891

**Published:** 2022-03-30

**Authors:** Hong Lv, Jing Yu, Hao Zhang, Xingjia Qian, Qian Wang, Bing Lu, Yifeng Sun

**Affiliations:** a Department of Pulmonary and Critical Care Medicine, Taicang Hospital of Chinese Medicine, Taicang City, Jiangsu Province, China; b Department of Pulmonary and Critical Care Medicine, Jiangsu Province Hospital of Chinese Medince, Nanjing, Jiangsu Province, China

**Keywords:** Bone marrow mesenchymal stem cell exosomes, MicroRNA-631, E2F family of transcription factor 2, non-small cell lung cancer

## Abstract

The exosomes (Exo) had always been considered as transport vectors for microRNA (miRNA). An increasing number of data had clarified the influence of Exo on the cell progression of non-small cell lung cancer (NSCLC). Nevertheless, its specific mechanism had not yet been verified. This work was to explore the potential mechanism of Exo-derived miR-631 targeting and regulating E2F family of transcription factor 2 (E2F2) to repress the malignant behavior of NSCLC cells. Test of microRNA (miR)-631 and E2F2 in NSCLC was performed. BMSCs-Exo that altered miR-631 was co-cultured with NSCLC cells. Detection of the cloning and progression of NSCLC cells was performed. Testification of the targeting of miR-631 with E2F2 was conducted. In *vivo* experiments were performed to verify the results in *vitro*. In short, elevation of miR-631 Exo repressed the advancement and phosphatidylinositol 3‐kinase/Akt activation of NSCLC cells, while silence of miR-631 was in the opposite. In terms of mechanism, miR-631 exerted the influence via targeting E2F2. The coincident results were obtained in animal models. In brief, BMSC-Exo mediated E2F2 via delivering miR-631 to NSCLC cells to modulate the malignant behavior of NSCLC.

## Graphical Abstract

**Figure:**
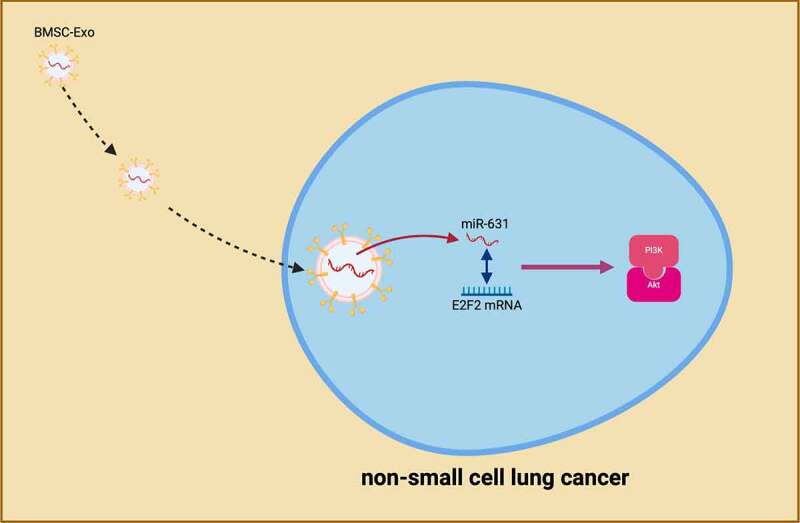

## 1 Introduction

Lung cancer (LC) is a prevalent malignant tumor [1]. A foregoing study has clarified that over 150,000 LC patients die worldwide each year, and 20,000 new cases are diagnosed [2]. Division of LC is into small cell lung cancer (SCLC) and non-small cell lung cancer (NSCLC), among of which NSCLC takes up about 85% of LC patients. The conventional subtypes of NSCLC are adenocarcinoma (AC), squamous cell carcinoma (SCC) and large cell carcinoma (LCC) [3]. Presently, surgical resection is the major treatment for NSCLC, and adoption of chemotherapy or radiotherapy is as adjuvant therapy [4]. Nevertheless, multiple patients with NSCLC are already diagnosed with advanced cancer, and limitation of drug treatment is frequently via the drug resistance of cancer cells [5]. Consequently, it is a necessity to seek for new treatments.

Bone marrow mesenchymal stem cells (BMSCs), non-hematopoietic progenitor cells, are able to be sucked from bone marrow with gene modification in *vitro*, offering a potential treatment strategy for cancer patients [6]. BMSCs-derived BMSCs-Exo is available to be adopted as gene vectors to deliver target genes to cancer cells, thereby repressing the malignant phenotype of cancer cells. Wang Y *et al*. clarify that microRNA (miRNA)-22-3p deriving from BMSC-Exo restrains the progression of colorectal cancer (CRC) via modulating the rhoptry associated protein (RAP) 2B/phosphatidylinositol 3‐kinase (PI3K)/Akt pathway [7]. Furthermore, miR-424 stemming from BMSC-Exo targets transforming growth factor type III receptor (TGFBR3) to boost CRC advancement [8]. Additionally, analogous functions of BMSC-Exo have also been testified in NSCLC. Studies have manifested that miR-144 and circular RNA (circRNA)-1395 deriving from BMSC-Exo is able to suppress the malignant phenotype of NSCLC [9,10]. Nevertheless, it is a necessity to further figure out the action of BMSC-Exo in NSCLC. MiRNA, a non-coding RNA, is elevated in the body [11]. MiRNA comprises a vital part post-transcriptional modulation of mRNA via stimulating the translational repression or degradation of its mRNA target [12]. MiR-631 is a kind of miRNA family, but few studies are involved for the moment. Furthermore, miR-631 is different in NSCLC [13]. Nevertheless, its biological function in NSCLC is yet unknown.

In this study, the biological function and potential molecular mechanism of miR-631 in NSCLC were explored. Additionally, whether BMSCs-Exo was able to deliver miR-631 to NSCLC to impact its malignant behavior was examined.

## 2 Method

### 2.1 Clinical sample collection

From 2010 to 2017, 48 pairs of NSCLC tissues and matching adjacent normal tissues were gained from Taicang Hospital of Chinese Medicine. Tumor specimens and corresponding adjacent normal tissue (≥5 cm) were collected and stored in liquid nitrogen or fixed in paraformaldehyde for subsequent analysis. E2F family of transcription factor 2 (E2F2) expression in the samples was examined by immunohistochemistry as previously described [14]. Patients who were pathologically diagnosed as NSCLC without accepting radiotherapy or chemotherapy were included. While patients were excluded for metastatic NSCLC, long-term adoption of glucocorticoid drugs and systemic infectious diseases and other diseases that was supposed to influence this study. All patients were informed consent, and authorization of the study was via the Medical Ethics Committee of Taicang Hospital of Chinese Medicine. This study was performed in line with the Declaration of Helsinki of the World Medical Association.

### 2.2 Cell culture

Culture of the purchased human normal bronchial epithelial cell (NBEC) line (BEAS-2B) and NSCLC cell line (H358, SPC-A-1, A549, H1299) (ATCC; the United States) was in Roswell Park Memorial Institute-1640 medium covering 100 U/mL (Gibco™; Thermo Fisher Scientific, Waltham, MA). Addition of 100 U/mL penicillin, 10% fetal bovine serum (FBS) and 100 U/mL streptomycin was conducted, and placing was in a humidified environment.

### 2.3 Cell transfection

Adoption of miR-631 mimics and inhibitors was for elevation or knockdown of miR-631, and augmented plasmids and small interfering RNAs (oe-E2F2 and si-E2F2) of targeting E2F2 were adopted for elevation or knockdown of E2F2. Additionally, adoption of the mimic NC, inhibitor NC, oe-NC and si-NC was as negative controls. All the above plasmids or oligonucleotides were purchased (all GenePharma, Shanghai, China). Placing of the A549 cells was in a 6-well plate for 24 h, and transfection of the above plasmids or oligonucleotides was into the cells adopting Lipofectamine 2000 (Thermo Fisher Scientific) in line with the manufacturer’s instructions [15]. After 48 h, adoption of the transfected cells was for follow-up studies.

### 2.4 Isolation and identification of BMSC

Separation of BMSCs was from the bone marrow of three donors via differential centrifugation [16]. Culture was in Dulbecco’s Modified Eagle Medium-F12 medium (Hyclone, South Logan, UT, USA) involving 10% FBS (10,099,141, Gibco, Carlsbad, CA, USA) and 0.2% penicillin and streptomycin. The passage was performed every 3 d, and selection of the 3^rd^ generation was for subsequent experiments.

Identification of the immunophenotype of BMSCs was performed adopting flow cytometry: Culture of the third generation of BMSC was to 80% confluence and separation was conducted. Count, centrifugation (2 × 10^5^ cells/tube) and suspension were performed in 1% bovine serum albumin (preparation was in 1 × phosphate buffer saline (PBS)). Accretion of the cell suspension (20 μL) was to polyethylene or fluorescein isothiocyanate (FITC) labeled monoclonal antibodies CD34, CD44, CD73 and CD90 (BD Company, USA), and reaction was in the dark for 30–60 min. Suspension of the cells was in 450 μL 1 × PBS, and detection was conducted adopting flow cytometer.

Identification of BMSCs adipogenic stimulation was performed: BMSCs were separated and calculated with 0.25% trypsin until the fusion was 80–90%. Seeding of the cells was in a 48-well plate at 7 × 10^3^ cells/well. Next, placing of the BMSCs was in an α-minimum essential medium (MEM) covering 10 μg/mL insulin, 1 μM dexamethasone, 10% FBS and 500 μM 3-isobutyl-1-methylxanthine. Shift of half the liquid in the 48-well plate every 2–3 d was conducted. After 14 d of stimulation, oil red O staining was performed. Fixation was with 300 μL 4% neutral formaldehyde solution for 30 min, staining was with 300 μL oil red O dye working solution, and protection from light for 15 min was performed. Ultimately, addition of 200 μL PBS was to the cells, and the morphology of lipid droplets was observed under an inverted microscope.

Identification of osteogenic stimulation of BMSCs was conducted: The third generation of BMSCs grew well with a fusion degree of 80–90%. The cells were gained via trypsin digestion. Seeding of the cell suspension in 7 × 10^3^ cells/well was into 48-well plates. Incubation of the cells was in α-MEM containing 0.05 mM vitamin C phosphate, 10–7 M dexamethasone, 10% FBS and 10 mM β-glycerophosphate, and the medium was updated in half every 2–3 d. After 14 d of stimulation, alkaline phosphtase (ALP) was performed. Fixation was with 200 μL fixative for 30 s, addition of 200 μL staining solution was performed and staining was for 30 min, and eventually, accretion of 200 μL PBS was conducted. ALP-positive cells were examined under an inverted microscope.

### 2.5 Transfection of BMSC

Seeding of BMSCs cells was at a concentration of 2 × 10^5^ cells/mL. After adherence, transient transfection of miR-631 mimic/inhibitor and the corresponding negative control (NC) was into BMSCs cells adopting Lipofectamine 2000 (Thermo Fisher Scientific), and the control treating with PBS was set up.

### 2.6 Separation and identification of Exo

Collection of the supernatant cultured via BMSCs was performed after transfection, and extraction of the Exo was conducted in line with the instructions of the exoEasy Maxi Kit, Qiagen). Observation of the size and morphology of Exo with a Jeol JEM1400 transmission electron microscope was performed (Jeol Ltd., Tokyo, Japan) [17]. Western blot analysis of Exo surface marker proteins CD9, CD81, CD63, tumor susceptibility gene 101 (TSG101) was performed.

### 2.7 Co-culture of the cell and Exo

Extraction of Exo was from BMSCs of transfecting with miR-631 mimic/inhibitor, and co-culture of Exo was with A549 cells of transfecting with miR-631 mimic/inhibitor or oe/si-E2F2 to explore the influence of Exo on the biological functions of NSCLC cells. After 48 h, collection of the cells was for subsequent studies.

### 2.8 Plate cloning

For analysis of cell colony formation, seeding of the transfected cells was into 6-well plates and culture was for 2 weeks. Furthermore, staining of the cell colonies was via adopting 0.1% crystal violet dye (J&L Biotech, Wuhan, Hubei). Ultimately, the photograph was taken adopting the XSP-22C microscope (Hu Xing, Pudong, Shanghai, China).

### 2.9 Flow cytometry detection of cell apoptosis

Seeding of 2 × 10^6^ cells/well was in a 6-well plate, separation was with 0.25% trypsin, and centrifugation of the cells was to remove the supernatant. Addition of 500 μL loading buffer, 10 μL propidium iodide solution and 5 μL Annexin V-FITC was performed in line with the instructions of Annexin V-FITC Apoptosis Detection Kit (K201-100, Biovision, USA), and reaction was for 20 min. The apoptosis rate was tested via flow cytometer (BD Biosciences).

### 2.10 Transwell migration and invasion

Determination of cell migration and invasion assays is carried out via a Transwell chamber (8 μm; Costar, Boston, MA, USA) [18]. Accretion of the transfected cells in a 24-well plate at a concentration of 5 × 10^4^ per well was to the upper chamber, and placing of the medium covering 10% FBS was in the bottom chamber. Adoption of the Transwell chamber was for the invasion assay with Matrigel (BD Biosciences, San Jose, CA, USA) package (Migration assay was not covered via Matrigel). After 24 h, fixation of the migrating or invasive cells was in 70% methanol (Sigma-Aldrich), and then staining was with 0.1% crystal violet (Sigma-Aldrich). Count of the cells was with a fluorescence microscope.

### 2.11 Reverse transcription quantitative polymerase chain reaction (RT-qPCR)

Extraction of total RNA was from cells or tissues adopting Trizol reagent (Invitrogen, Carlsbad, California, USA) in line with the manufacturer’s instructions. Reverse transcription was performed adopting the first-strand cDNA synthesis kit (Takara, Otsu, Shiga, Japan). RT-qPCR was conducted exerting the iCycler IQ multicolor detection system (Bio-Rad, Hercules, California, USA) with IQ SYBR Green Supermix (Bio-Rad). Calculation of gene was via 2^−ΔΔCt^ method. Adoption of glyceraldehyde-3-phosphate dehydrogenase (GAPDH) or U6 was as internal reference genes for mRNA and miRNA, separately. The primer sequence is presented in Table 1.

**Table 1.** PCR primer sequences

	Primer sequences (5ʹ – 3ʹ)
MiR-631	Forward: 5’- TCTCTGGGCCTGTGTCTTAGGC-3’
	Reverse: 5’- TTAATGGGGTGATTGGTGGT-3’
E2F2	Forward: 5’- CAGCAGTAGCAGCAGCGTAGTC-3’
	Reverse: 5’- CCATCCGACGACATCTCCAAGC-3’
GAPDH	Forward: 5’- CTGCCAACGTGTCAGTGGTG-3’ Reverse: 5’- TCAGTGTAGCCCAGGATGCC-3’
U6	Forward: 5’- CGAATTTGCGTGTCATCCTT-3’
	Reverse: 5’- CGAATTTGCGTGTCATCCTT-3’

### 2.12 Western blot

Extraction of the protein sample was with radio-immunoprecipitation assay lysis buffer, and measurement of the concentration was via the Bicinchoninic acid method. A total of 20 g samples were applied to 12% sulfate-polyacrylamide gel, electrophoresis was performed to separate proteins, and then electroblot was onto polyvinylidene fluoride membrane (Invitrogen, Carlsbad, CA, USA), which was sealed with 5% skimmed milk powder for 1 h. Subsequently, incubation of the membrane was with the following primary antibodies: CD9 (ab92726, Abcam), E2F2 (14,129, 1:1000, Cell Signaling Technology), CD81 (ab79559, Abcam BD Biosciences), CD63 (ab134045, Santa Cruz Biotechnology), TSG101 (ab125011, GAPDH (2118, Cell Signaling Technology) and Santa Cruz Biotechnology). Incubation was with secondary antibodies (1:5000; Abcam, Cambridge, UK) conjugating to horseradish peroxidase at the temperature for 1 h. After exposure to enhanced chemiluminescence reagents (Amersham Biosciences, Fairfield, CT, USA), collection of images was performed adopting a gel imaging system with ImageJ software for analyzing protein band gray values.

### 2.13 The luciferase reporter experiment

Prediction of the binding site of miR-631 and E2F2 was exerting bioinformatics software https://cm.jefferson.edu/rna22/, and generation of wild-type (WT) E2F2 vector (E2F2-WT) and mutant-type (MUT) E2F2 vector (E2F2-MUT) was via PCR amplification adopting QuikChange site-directed mutagenesis kit (Stratagen, Santa Clara, USA). Co-transfection of 100 ng WT or MUT E2F2 luciferase vector with 100 nM miR-631-mimic (RiboBio, Guangzhou, China) or 100 nM negative control (NC-mimic) (RiboBio, Guangzhou, China) was into A549 cells adopting LipofectamineTM 2000 (Thermo Fisher Scientific) in line with the manufacturer’s requirements. After transfection of 48 h, luciferase was determined adopting the dual luciferase reporter gene assay system (Promega).

### 2.14 Animal experiment

Authorization of all animal experiments was via the Animal Ethics Committee of Taicang Hospital of Chinese Medicine. Thirty-six BALB/c nude mice of 4 weeks weighing approximately 10–12 g were performed (all Hunan SJA Laboratory Animal Co., Ltd., Changsha, China). Animals are raised in a specific pathogen-free (SPF) grade environment. Injection of the A549 cell suspension (1 × 10^7^ cells/mouse) was subcutaneously into each mouse to construct a subcutaneous xenograft tumor model. Meanwhile, injection of Exo secreted via BMSCs (100 μL at a concentration of 1 μg/μL) was into the tail vein of each mouse on days 5, 10, 15 and 20 until the average tumor volume was 100 mm^3^. Injection cover: normal saline (the control), Exo without any oligonucleotides (the Exo), Exo carrying miR-631 mimic (the Exo-miR-631 mimic), and mimic NC-carrying Exo (the Exo-mimic NC), Exo carrying miR-631 inhibitor (the Exo-miR-631 inhibitor), Exo carrying inhibitor NC (the Exo-inhibitor NC). Measurement of the tumor volume was with vernier calipers every 5 d. Tumor volume = 0.5 × (long axis × short axis) ^2^. After 25 d, euthanasia of all nude mice was performed and separation of their tumor tissues was for Western blot analysis.

### 2.15 Statistical analysis

Data analysis was adopting SPSS21.0 (IBM Corporation, Armonk, NY, USA). Representation of the measurement data was as mean ± standard deviation (SD). Differences between groups were adopting t test, comparisons between groups were exerting one-way analysis of variance (ANOVA), and Tukey’s multiple comparison test was performed after analysis of variance. Testification of the association of miR-631 with the clinicopathological features of NSCLC patients was exerted. *P* < 0.05 was accepted as indicative of significant differences.

## 3 Results

### 3.1 MiR-631 is silenced in NSCLC

**Figure 1.**
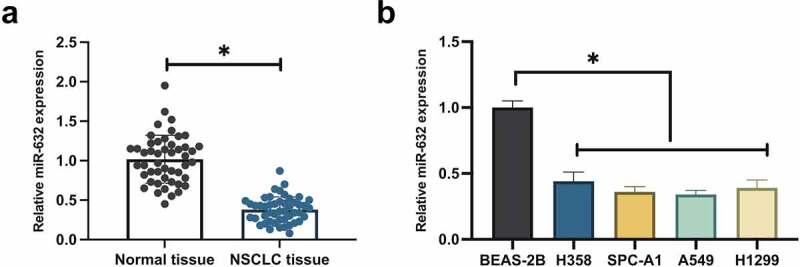
MiR-631 is silenced in NSCLC.

MiR-631 in NSCLC was figured out to examine the influence of miR-631 on the malignant behavior of NSCLC. MiR-631 was decline in NSCLC tissues, as presented in Figure 1a. Subsequently, miR-631 in NSCLC cell lines were examined. The results clarified that miR-631 in NSCLC cell lines (H358, SPC-A-1, A549, H1299) was distinctively declined versus the human NBEC line (BEAS-2B), and A549 cells manifested the most declined miR-631 (Figure 1b). Additionally, division of NSCLC patients was into the elevated and the declined in line with the median miR-631, and the association of miR-631 with clinicopathological features was analyzed. The results clarified (Table 2) that miR-631 was distinctively associated with tumor size in tumor node metastasis (TNM) staging, but which was not correlated with age and gender, histological grade, and smoking history. In general, miR-631 was declined in NSCLC and was correlated with the clinicopathological features of NSCLC patients.

**Table 2.** Correlation is of miR-631 with clinicopathological features

Features	Groups	Cases	MiR-631		*P*
			The elevated (n = 24)	The declined (n = 24)	
Age	<60	15	7	8	0.7555
	≥60	33	17	16	
Gender	Male	29	17	12	0.1400
	Female	19	7	12	
Tumor size (cm)	≥5 cm	38	15	23	0.0045
	<5 cm	10	9	1	
Histological grade	Moderate	30	13	17	0.2330
	Low	18	11	7	
TNM staging	I + II	22	14	8	0.0822
	III + IV	26	10	16	
Smoking history	Smoking	35	17	18	0.7453
	No smoking	13	7	6

A. Detection of miR-631 in NSCLC tissues was via RT-qPCR; B. Test of miR-631 in NSCLC cell lines and human normal bronchial epithelial cell lines was via RT-qPCR; The values were average ± standard deviation (SD) (B, N = 3). * *P* < 0.05.

### 3.2 Elevation of miR-631 was conducive to repress the malignant behavior of NSCLC cells

**Figure 2.**
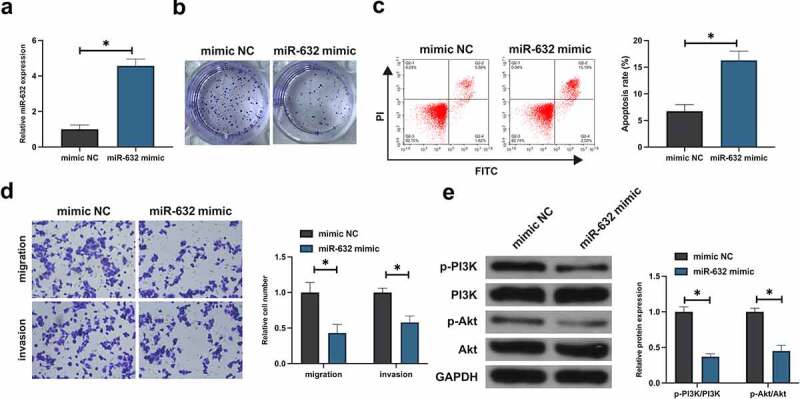
Elevation of miR-631 is conducive to repress the malignant phenotypes in NSCLC cells.

Subsequently, the impact of miR-631 on the malignant behavior of NSCLC was figured out. After transfection with miR-631-mimic, miR-631 was distinctly augmented in A549 (Figure 2a). Besides, the viability of A549 cells was distinctively declined after elevation of miR-631 (Figure 2b). Augmentation of miR-631 repressed A549 cell advancement (Figure 2c, D). Subsequently, the impact of elevation of miR-631 on the PI3K/Akt signal pathway of A549 cells was tested. Elevation of miR-631 dramatically declined the phosphorylation of PI3K/Akt, as presented in Figure 2e. In short, elevation of miR-631 was available to repress the malignant behavior of NSCLC and inactivate the PI3K/Akt pathway.

A. Examination of miR-631 was via RT-qPCR; B: Detection of cell proliferation was via plate cloning; C. Test of cell apoptosis was via Flow cytometry; D. Examination of cell migration and invasion was via Transwell; E. Test of cell PI3K/Akt Phosphorylation was via Western blot. Presentation of values was with the mean ± SD (n = 3), and the distinction of each group was calculated via ANOVA, and the variance was corrected via Tukey’s test, *P < 0.05.

### 3.3 E2F2 is the target gene of miR-631, and elevated E2F2 facilitates the malignant behavior of NSCLC

**Figure 3.**
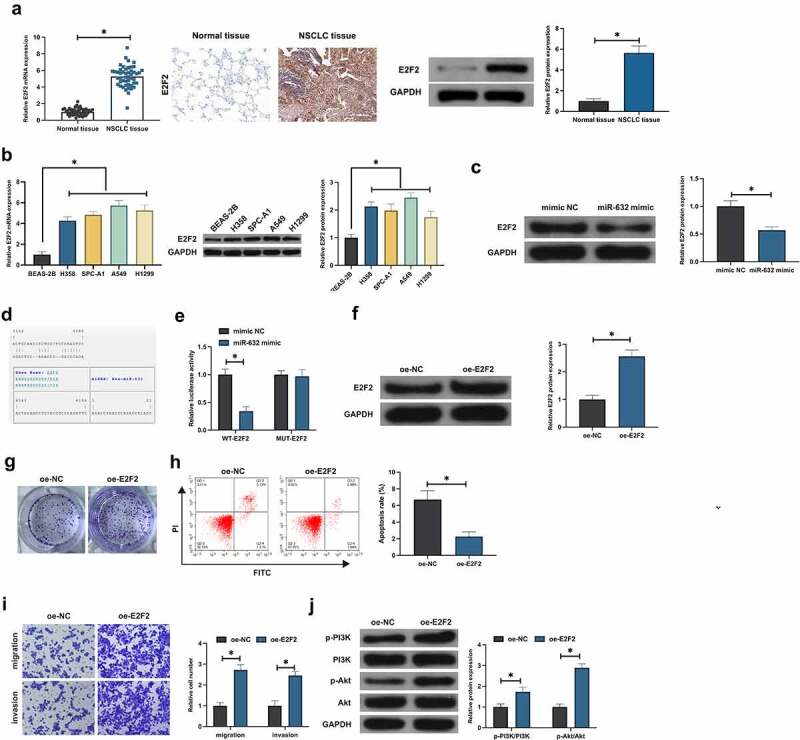
E2F2 is the target gene of miR-631, and elevated E2F2 facilitates the malignant behavior of NSCLC.

E2F2 was testified to perform as an oncogene in multiple cancers [19]. RT-qPCR, Western blot and IHC manifested E2F2 in NSCLC tissues and adjacent normal tissues and the cells were examined, which were elevated in NSCLC (Figure 3a, b). Notably, E2F2 was dramatically declined in A549 cells of elevation for miR-631 (Figure 3c). Consequently, correlation was supposed to be of miR-631 with E2F2. Furthermore, miR-631 and ZEB2 were provided with targeting (Figure 3d). Additionally, co-transfection of E2F2-WTand miR-631 mimic luciferase activity was distinctly declined after co-transfection with E2F2-WTand miR-631 mimic, while the luciferase activity was not critically altered after co-transfection with E2F2-WTand miR-631-mimic (Figure 3e), which clarified that E2F2 was the target gene of miR-631. Subsequently, the biological function of E2F2 in NSCLC was tested. E2F2 was elevated in A549 cells via transfecting oe-E2F2 (figure 3f). After augmentation of E2F2, the A549 cell advancement was dramatically accelerated (Figure 3g-I). Additionally, elevation of E2F2 facilitated the phosphorylation of PI3K/Akt (Figure 3j). In brief, E2F2 was a target gene of miR-631 and performed as an oncogene in NSCLC.

A. Test of E2F2 expression in NSCLC and normal tissues was via RT-qPCR, Western blot and IHC; B. Examination of E2F2 in NSCLC cell lines and human normal bronchial epithelial cell lines was via RT-qPCR and Western blot; C. Test of E2F2 in A549 cells after elevation of miR-631 was via Western blot; D. Binding sites at Bioinformatics website; Prediction of miR-631 and E2F2 was via https://cm.jefferson.edu/rna22/ andE. Analysis of the targeting of miR-631 with E2F2 was with the luciferase experiment; F: Examination of E2F2 in A549 cells after augmentation of E2F2 was via Western blot; G. Test of A549 cell proliferation after elevation of E2F2 was via plate clone; H. Examination of A549 cell apoptosis after augmentation of E2F2 was via flow cytometry; I. Test of A549 cell invasion and migration after elevation of E2F2 was via Transwell; J. Examination of the phosphorylation of PI3K/Akt in A549 cells after elevation of E2F2 was via Western blot. The value was presented as mean ± SD (n = 3). * *P* < 0.05.

### 3.4 MiR-631 exerts an influence on the malignant behavior of NSCLC via modulating E2F2

**Figure 4.**
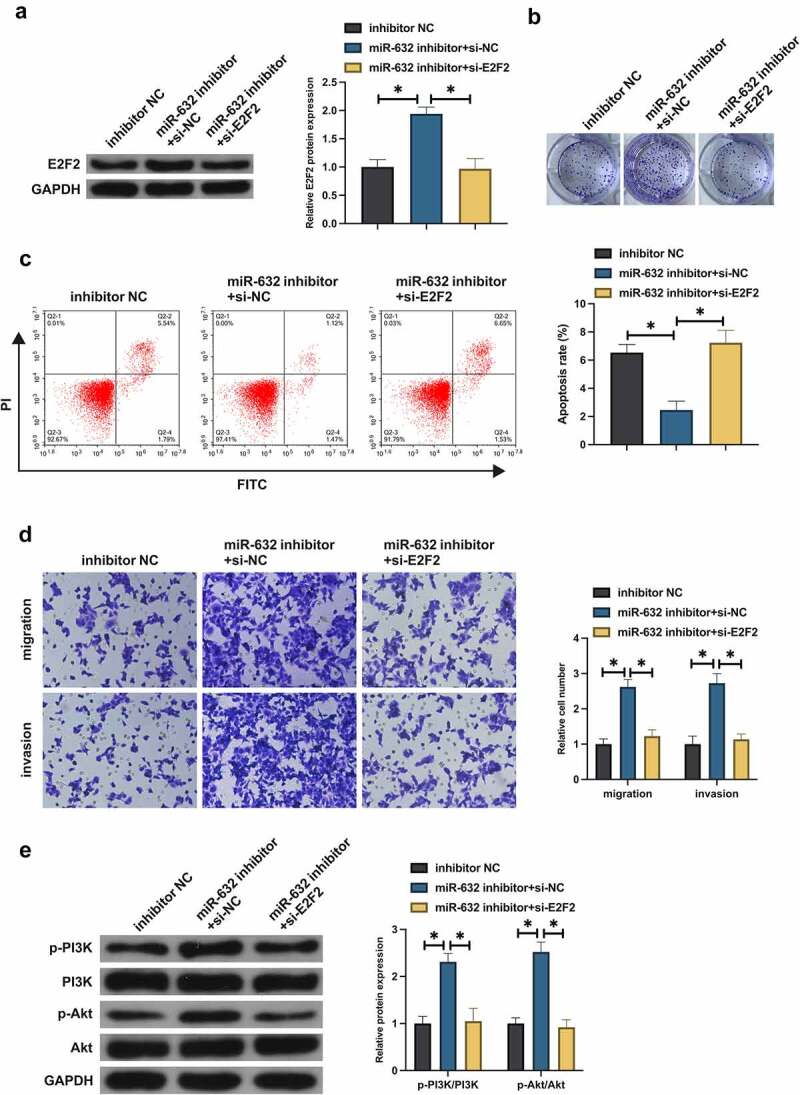
MiR-631 exerts an influence on the malignant behavior of NSCLC via modulating E2F2.

Whether miR-631 impacted the malignant behavior of NSCLC via regulating E2F2 was figured out. Co-transfection of the miR-631 inhibitor and si-E2F2 was into A549 cells. Transfection of miR-631 inhibitor elevated E2F2, but this phenomenon was prevented after co-transfection of si-E2F2 (Figure 4a). Silence of miR-631 accelerated A549 cell advancement, but decline of E2F2 turned around A549 cell progression (Figure 4b, D). Furthermore, silence of miR-631 declined A549 cell apoptosis, but decline of E2F2 recovered A549 cell apoptosis (Figure 4c). It manifested that miR-631 inhibited malignant behavior of NSCLC by targeting E2F2 expression.

A. Examination of E2F2 after co-transfection was via Western blot; B: Detection of A549 cell proliferation after co-transfection was via plate cloning; C. Test of A549 cell apoptosis after co-transfection was via Flow cytometry; D. Examination of A549 cell migration and invasion after co-transfection was via Transwell; E. Test of Phosphorylation of PI3K/Akt in A549 cell after co-transfection was via Western blot; Values were presented as mean ± SD (N = 3). * *P* < 0.05.

### 3.5 Identification of BMSCs and Exo

**Figure 5.**
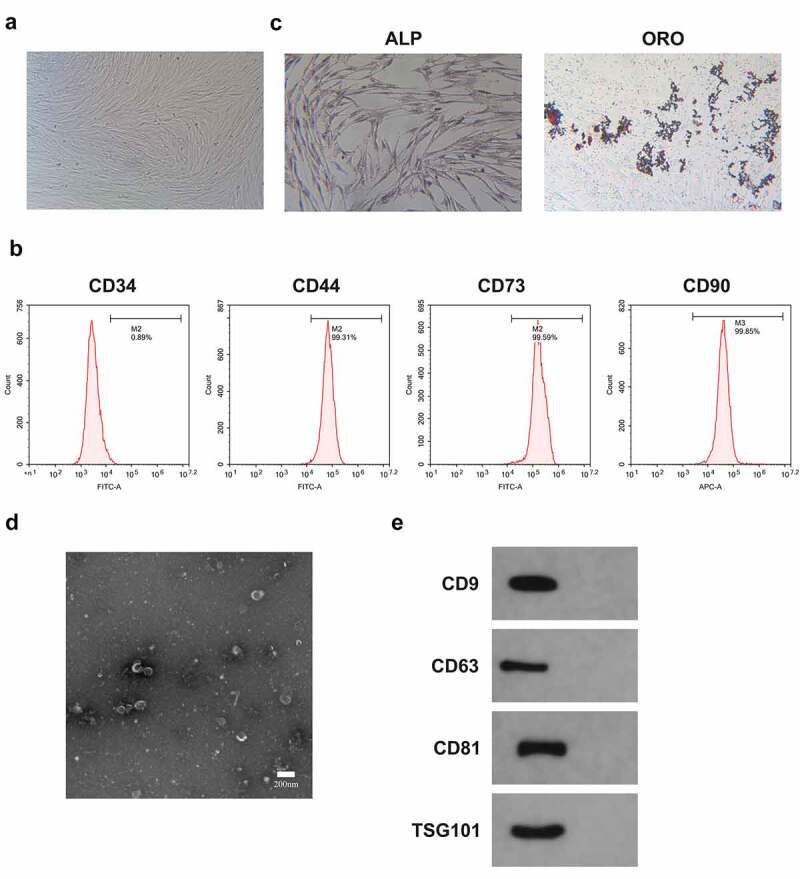
Identification of BMSCs and Exo.

Many studies have manifested BMSCs-Exo delivery of miRNA to recipient cancer cells has a positive effect on inhibiting oncogenesis [20] [PMID: 32,978,367]. It was hypothesized that BMSCs-Exo could deliver miR-631 to A549 cells and affect their growth. BMSCs were first isolated from bone marrow. Observation of the isolated BMSCs exhibited a fusiform fibrous morphology was via an inverted microscope (Figure 5a). Furthermore, CD44, CD73, and CD90 in the separated BMSCs were positive, while CD34 was negative (Figure 5b). Meanwhile, the isolated cells were provided with typical features of BMSCs and were able to be stimulated into osteogenisis or adipogenesis, which was in agreement with foregoing reports [5] (Figure 5c). Consequently, BMSCs were successfully separated.

A. Observation of the morphology of BMSCs with an inverted microscope; B. Test of BMSCs surface markers CD44, CD73, CD34 and CD90 was via flow cytometry; C. ALP staining and Oil-Red-O staining to Examination of ALP activity and lipid after BMSCs osteogenic or adipogenic induction was via ALP staining and Oil-Red-O staining; D. Observation of Exo morphology was via TEM; E. Test of Exo-labeled proteins CD9, CD63, CD81 and TSG101 was via Western blot.

Subsequently, Exo was isolated from BMSCs cells. Furthermore, the Exo deriving from BMSCs was characterized and analyzed. The results of transmission electron microscopy (TEM) clarified that mesenchymal stem cell-derived exosomes (MSC-Exo) were spherical, and their diameters primarily ranged from 40 nm to 110 nm (Figure 5d). Besides, these nanovesicles express exosomal marker proteins like CD63, CD9, TSG101 and CD81 (Figure 5e). Consequently, Exo was successfully separated from BMSCs.

### 3.6 BMSCs-Exo exerts an influence on the malignant behavior of NSCLC via the miR-631/E2F2 axis

**Figure 6.**
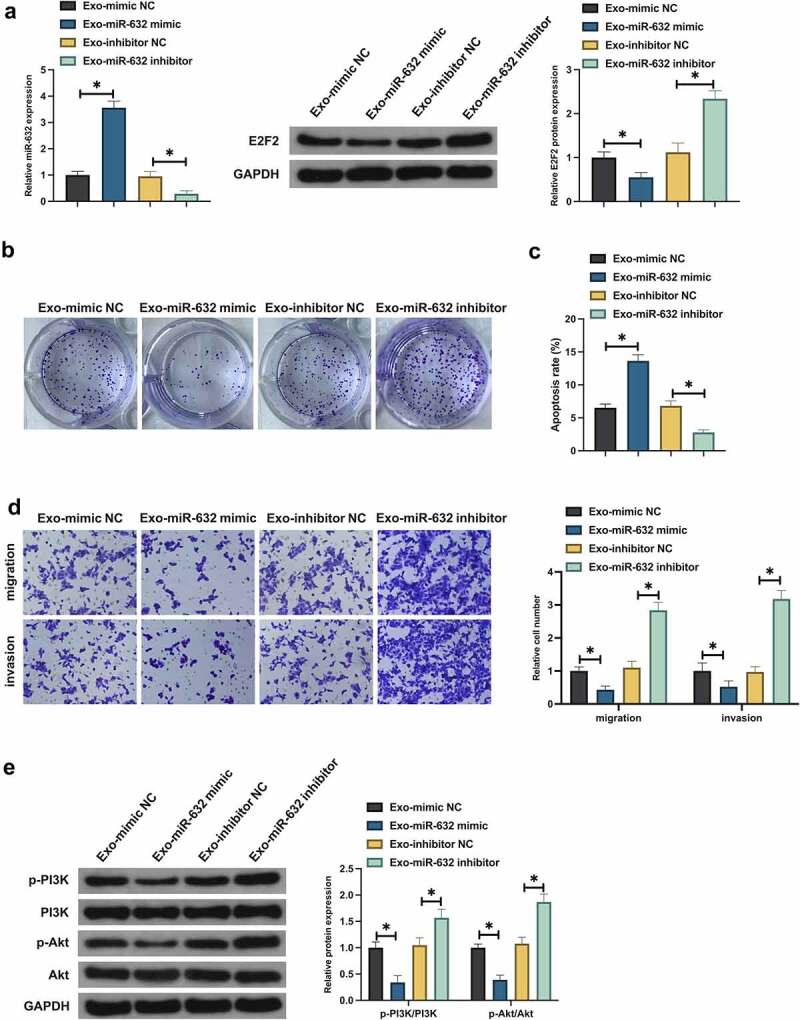
BMSCs-Exo exerts an influence on the malignant behavior of NSCLC via the miR-631/E2F2 axis.

Whether Exo impacted the malignant behavior of NSCLC via miR-631/E2F2 axis was tested. Primarily, whether Exo was able to deliver miR-631 to A549 cells was examined. After co-culture with Exo carrying miR-631 mimic, miR-631 was elevated, but E2F2 was declined in A549 cells, while miR-631 inhibitor was in the opposite (Figure 6a). And whether miR-631 in Exo exerted an influence on the biological progress of NSCLC resistant cells was tested. Co-culture with Exo carrying miR-631 mimic distinctively repressed A549 cell advancement and activation of PI3K/Akt signaling pathway, while co-culture with Exo carrying miR-631 inhibitor facilitated malignant behavior of A549 cells (Figure 6b-E). In general, BMSCs-Exo exerted an influence on the malignant behavior of NSCLC via delivering miR-631 and mediating E2F2.

A. Examination of miR-631 and E2F2 in A549 cells after co-culture carrying miR-631 mimic/inhibitor Exo was via RT-qPCR and Western blot; B: Detection of A549 cell proliferation after co-culture carrying miR-631 mimic Exo was via plate cloning; C. Test of A549 cell apoptosis after co-culture carrying miR-631 mimic Exo was via flow cytometry; D. Examination of A549 cell migration and invasion after co-culture carrying miR-631 mimic Exo was via Transwell; E. Test of Phosphorylation of PI3K/Akt in A549 cell after co-culture carrying miR-631 mimic Exo was via Western blot; Values were presented as mean ± SD (N = 3).* *P* < 0.05.

### 3.7 Exo impacted tumor growth via miR-631/E2F2 pathway

**Figure 7.**
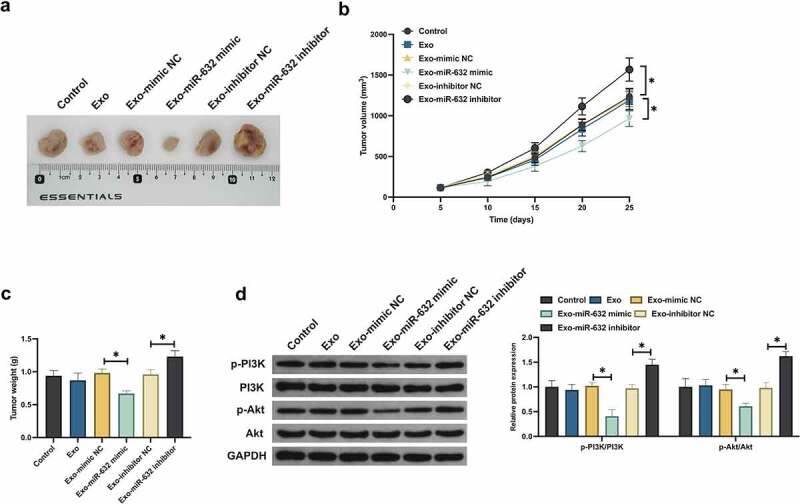
Exo-derived miR-330p-5p impacts tumor cisplatin sensitivity in *vitro.*

Ultimately, verification of the results of the above cell experiments via animal model experiments was conducted to testify its rationality. Exo did not decline the tumor volume and weight, but which was declined with Exo carrying miR-631 mimic (Figure 7a-C). Additionally, Exo carrying miR-631 inhibitor elevated the tumor volume and weight. Furthermore, Exo exerted no influence on E2F2, PI3K and Akt in tumor tissues. Exo carrying miR-631 mimic declined E2F2 and phosphorylation of PI3K/Akt in tumor tissues, while Exo carrying miR-631 inhibitor was in the opposite (Figure 7d). In short, Exo silenced E2F2 in tumor tissues via transporting miR-631 to restrain NSLCL tumor advancement.

A: Representative tumor pictures; B. Mouse tumor volume growth curve; C. Mouse tumor weight; D. Test of tumor E2F2, PI3K and Akt was via Western blot. The value was presented as mean ± SD (n = 6). * *P* < 0.05.

## 4 Discussion

NSCLC is the most prevalently diagnosed cancers and the vital cause of cancer deaths worldwide as well [20]. Exo deriving from BMSCs has been testified to have profitable functions in ameliorating cancer proliferation, metastasis and angiogenesis. In this study, the role and potential molecular mechanism of BMSCs-Exo in NSCLC was further uncovered. The results elucidated that BMSCs-Exo down-regulated E2F2 and reduced the phosphorylation of PI3K/Akt via delivering miR-631 to A549 cells. The variation of these genes was in favor of repressing A549 cell proliferation, invasion, migration and promoting apoptosis.

In the research, miR-631 was founded to be distinctively silenced in NSCLC patient tissues and NSCLC cell lines. Foregoing studies have elucidated that miR-631 is silenced in NSCLC tissues, and the clinical diagnosis of miR-631 is available to be adopted as a potential biomarker for NSCLC [13]. This viewpoint was further supported with the results of this study. Additionally, miR-631 is founded to be correlated with TNM staging and differentiation of NSCLC patients. TNM staging is the determinants of cancer staging system and disease prognosis. As reported, declined miR-631 is also highly associated with poor tumor differentiation and TNM staging of LC [21]. The vital role of miR-631 in the clinical diagnosis of cancer was further strengthened. Foregoing studies have manifested that elevation of miR-631 suppresses prostate cancer and LC’s proliferation, invasion and migration [21,22]. The results of the study indicated miR-631 also manifested an analogous action in NSCLC, clarifying that miR-631 is a tumor suppressor gene. Additionally, foregoing studies have also stressed the critical function of miR-631 in cancer epithelial-mesenchymal transition (EMT), chemoresistance and distant metastasis [21,23]. Consequently, it is a necessity to further figure out the action of miR-631 in EMT, chemoresistance and distant metastasis of NSCLC in subsequent studies.

In the study, the luciferase activity founded miR-631 targeted E2F2 in NSCLC. E2F2, a prevalent oncogene, modulates extensive biological processes, covering cell cycle, DNA damage responses and apoptosis. As reported, elevated E2F2 is able to facilitate NSCLC proliferation, invasion, migration and inhibit apoptosis [24–27]. It was in agreement with the results of this study. Additionally, the results of the research manifested miR-631 prevented the activation of PI3K/Akt signaling pathway in NSCLC via targeting E2F2. Multiple studies have reported the function of E2F2 in modulating cancer PI3K/Akt signaling. Li H *et al*. find that E2F2 accelerates autophagy in gastric cancer via modulating the PI3K/Akt/mammalian target of rapamycin (mTOR) pathway [28]. The latest study has elucidated that E2F2 drives glioma’s progression via the PI3K/Akt pathway in a 6-phosphofructo-2-kinase/fructose-2,6-biphosphatase 4 (PFKFB4)-dependent manner [29]. The results of the study further affirmed the vital role of E2F2 in modulating PI3K/Akt signal.

BMSCs, a group of pluripotent stem cells, are available to differentiate into chondrocytes, adipocytes and osteoblasts [30]. Multiple studies have manifested that Exo secreted via BMSCs are able to be adopted as vectors for modifying genes or drugs [31]. The gene in the Exo after being delivered to the recipient cells exerts a critical influence on the modulation of gene of the recipient cells, thereby modulating the biological behavior of the recipient cells [32]. The results of this study indicated BMSCs-Exo silenced E2F2 in NSCLC via delivering miR-631 to NSCLC cells to repress the malignant behavior of NSCLC, which was verified in *vivo* experiments. Previous studies have demonstrated BMSCs-Exo affects cancer malignant phenotypes by delivering miRNAs into NSCLC cells. MiR-204 [33], miR-193a [5] and miR-144 [10] are included in these miRNAs. The data from this study further complement and support the role of Exo in delivering miRNA [34].

## 5 Conclusion

In general, miR-631 modulated the PI3K/Akt pathway via targeting E2F2 to repress the malignant behavior of NSCLC. Exo deriving from BMSCs were available to deliver miR-631 to NSCLC to exert an analogous action. These findings offered a new conciseness for the targeted treatment of NSCLC. Nevertheless, the action of BMSCs only in animal models and cells was testified in this study, and it was yet unknown how effective its clinical application was, which needed to be further determined in subsequent studies.
